# Indoxyl-Sulfate-Induced Redox Imbalance in Chronic Kidney Disease

**DOI:** 10.3390/antiox10060936

**Published:** 2021-06-09

**Authors:** Chien-Lin Lu, Cai-Mei Zheng, Kuo-Cheng Lu, Min-Tser Liao, Kun-Lin Wu, Ming-Chieh Ma

**Affiliations:** 1 Division of Nephrology, Department of Medicine, Fu Jen Catholic University Hospital, New Taipei 24352, Taiwan; janlin0123@gmail.com; 2 School of Medicine, Fu Jen Catholic University, New Taipei 242062, Taiwan; 3 Division of Nephrology, Department of Internal Medicine, College of Medicine, Taipei Medical University, Taipei 11031, Taiwan; 11044@s.tmu.edu.tw; 4 Division of Nephrology, Department of Internal Medicine, Taipei Medical University Shuang Ho Hospital, New Taipei 23561, Taiwan; 5 Research Center of Urology and Kidney, Taipei Medical University, Taipei 11031, Taiwan; 6 Division of Nephrology, Department of Medicine, Taipei Tzu Chi Hospital, Buddhist Tzu Chi Medical Foundation, New Taipei 23142, Taiwan; kuochenglu@gmail.com; 7 Department of Pediatrics, Taoyuan Armed Forces General Hospital, Taoyuan 32551, Taiwan; liaoped804h@yahoo.com.tw; 8 National Defense Medical Center, Department of Pediatrics, Tri-Service General Hospital, Taipei 114202, Taiwan; 9 Division of Nephrology, Department of Internal Medicine, Taoyuan Armed Forces General Hospital, Taoyuan 32551, Taiwan

**Keywords:** AST-120, chronic kidney disease, indoxyl sulfate, oxidative stress

## Abstract

The accumulation of the uremic toxin indoxyl sulfate (IS) induces target organ damage in chronic kidney disease (CKD) patients, and causes complications including cardiovascular diseases, renal osteodystrophy, muscle wasting, and anemia. IS stimulates reactive oxygen species (ROS) production in CKD, which impairs glomerular filtration by a direct cytotoxic effect on the mesangial cells. IS further reduces antioxidant capacity in renal proximal tubular cells and contributes to tubulointerstitial injury. IS-induced ROS formation triggers the switching of vascular smooth muscular cells to the osteoblastic phenotype, which induces cardiovascular risk. Low-turnover bone disease seen in early CKD relies on the inhibitory effects of IS on osteoblast viability and differentiation, and osteoblastic signaling via the parathyroid hormone. Excessive ROS and inflammatory cytokine releases caused by IS directly inhibit myocyte growth in muscle wasting via myokines’ effects. Moreover, IS triggers eryptosis via ROS-mediated oxidative stress, and elevates hepcidin levels in order to prevent iron flux in circulation in renal anemia. Thus, IS-induced oxidative stress underlies the mechanisms in CKD-related complications. This review summarizes the underlying mechanisms of how IS mediates oxidative stress in the pathogenesis of CKD’s complications. Furthermore, we also discuss the potential role of oral AST-120 in attenuating IS-mediated oxidative stress after gastrointestinal adsorption of the IS precursor indole.

## 1. Introduction

The presence of oxidative stress is a result of the imbalance between the increase in reactive oxygen/nitrogen species (ROS/RNS) and the decrease in antioxidant capacities (redox imbalance). Oxidative stress has a devastating effect on cellular carbohydrates, lipids, proteins, and nucleic acids, which hinders normal physiological functions. Oxidative stress is a well-known causative factor in developing atherosclerotic lesion formation, which contributes to cardiovascular (CV) disease [1,2]. In chronic kidney disease (CKD), such oxidative-stress-related redox imbalance is linked to the pathological complications caused by the accumulation of uremic toxins, such as CV disease, renal function decline, uremic bone disease, muscle wasting, and renal anemia [3,4,5,6,7,8]. Uremic toxins are compounds that are usually excreted by the kidneys, and accumulate in the blood during the progression of CKD. Uremic toxins can be classified by a useful and widely accepted categorization based on plasma-protein-binding characteristics and molecular weight: low (<500 daltons), middle, and high (>12,000 daltons) [9]. Low-molecular-weight, water-soluble compounds lack protein-binding capacity, and are quite easily removed by conventional hemodialysis. Low-molecular-weight, water-soluble compounds include urea, creatinine, phosphate, guanidine, and asymmetric dimethylarginine. Urea and creatinine do not exert much toxicity on physiological functions, and are commonly used as markers for assessing renal function and dialysis clearance [10]. Conversely, protein-bound uremic toxins are hard to remove via dialysis due to their protein-binding capacity, even though their molecular weight is less than 500 daltons. Most of these protein-bound uremic toxins are generated by intestinal bacteria fermentation, and include phenolic compounds originated from tyrosine and phenylalanine, as well as indolic compounds originated from tryptophan, such as indoxyl sulfate (IS) [11]. Both of these compounds might be involved in the pathogenesis of accelerated CV disease and mortality in CKD [12,13,14]. The involvement of uremic toxins in redox imbalance has recently provoked a growing interest in CKD. In fact, CKD patients typically suffer from redox imbalance associated with uremic toxins, which often worsens CKD complications progressively with the degree of renal failure. Accumulating evidence has shown that IS exhibits pro-oxidant effects on various exposed tissues in CKD [15,16]. Thus, investigating IS’s pro-oxidant role is of primary importance in CKD-associated complications. This article summarizes the mechanisms by which IS exerts pro-oxidant influence in CKD patients, and how AST-120, an oral charcoal adsorbent, facilitates IS accumulation and ameliorates IS-mediated organ damage in CKD.

## 2. Overview of the Uremic Toxin Indoxyl Sulfate

**Figure 1:**
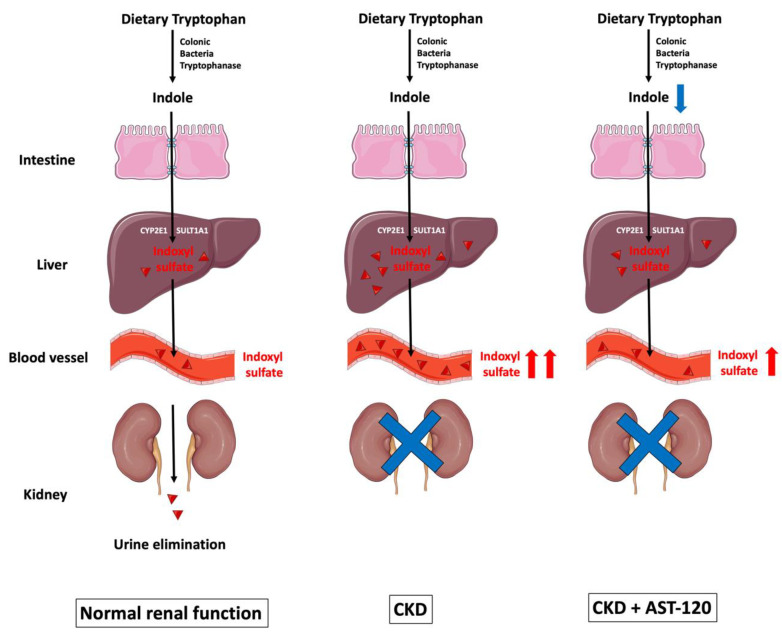
The biological formation of indoxyl sulfate. Dietary tryptophan is converted to indole by tryptophanase-expressing colonic bacteria, and is then absorbed across the intestinal epithelial cells. Reduced expression of tight junction proteins—such as claudin-1, occludin, and ZO-1—facilitates indole entry. Once indole is uptaken by hepatocytes, it is metabolized by the hepatic microsomal CYP450 enzymes CYP2E1 and SULT1A1 to form IS, which is eventually eliminated by the kidneys via tubular secretion. As CKD progresses, IS gradually accumulates in circulation because of reduced renal excretion. AST-120 is a charcoal adsorbent that adsorbs indole production in the gut, and subsequently decreases serum IS accumulation caused by CKD. CKD: chronic kidney disease; CYP2E1: cytochrome P450 2E1; IS: indoxyl sulfate; SULT1A1: sulfotransferase 1A1; ZO-1: zonula occludens-1. Parts of the figure were drawn by using pictures from Servier Medical Art. Servier Medical Art by Servier is licensed under a Creative Commons Attribution 3.0 Unported License (https://creativecommons.org/licenses/by/3.0/).

Among the aforementioned uremic retention solutes, 27.8% are protein bound [9]. IS belongs to the indole group of uremic toxins, and is a protein-bound, low-molecular-weight solute with a molecular weight of 213.21 daltons. Indole-group uremic toxins also comprise indole-3-acetic acid, kynurenine, kynurenic acid, and quinolinic acid, and these are all protein-bound solutes [17]. As shown in Figure 1, tryptophanase-expressing bacteria in the lumen of the gut convert dietary tryptophan into indole, which is then absorbed across the intestinal epithelial cells. The tight junction proteins—such as claudin-1, occludin, and zonula occludens-1 (ZO-1)—located in the gut epithelial cell membrane are known for their barrier-forming abilities, and are used to seal the gaps between gut epithelial cells. In CKD, decreased expression of these tight junction proteins disrupts the integrity of the gut epithelium, facilitating the entry of uremic toxins [18].

Once indole is absorbed across the intestinal epithelial cells into the blood, it is uptaken by hepatocytes; this can be metabolized by the hepatic microsomal CYP450 enzyme CYP2E1 and the sulfotransferase family 1A member 1 (SULT1A1) to form IS, which is eventually removed by the kidneys via tubular secretion [19,20,21]. Cytochrome P450 family 2 subfamily E member 1 (CYP2E1) is a phase I detoxification enzyme within the liver, and is responsible for metabolizing ethanol and catalyzing the 3-hydroxylation of indole in hepatic microsomes [19]. Subsequently, 3-hydroxyindole (indoxyl) is sulfated by SULT1A1 to form 3-indoxylsulfate and 3-indoxylsulfuric acid (also known as IS) in the liver [20].

AST-120, an oral charcoal adsorbent, is therapeutically used to lower serum IS levels in CKD patients [22]. AST-120 not only adsorbs indole produced by gastrointestinal tract bacteria, but also ameliorates IS-mediated cell damage in CKD through the suppression of oxidative stress [23].

**Figure 2:**
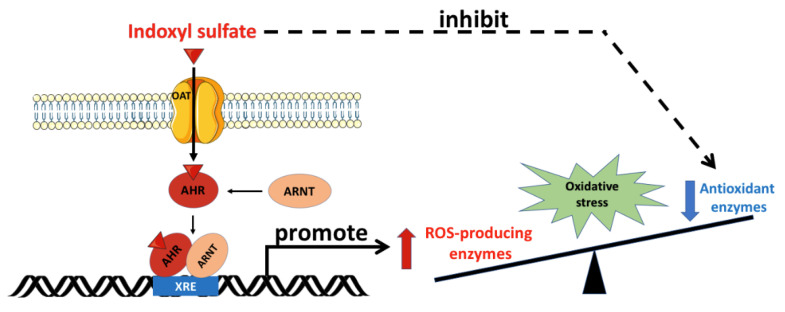
Oxidative stress mediated by indoxyl sulfate. OATs mediate the uptake of IS into the cell, and IS binds to the cytoplasmic AhR, which subsequently translocates into the nucleus. In the nucleus, the IS/AhR complex dimerizes with ARNT as a transcriptional activator by binding with the XRE sequence in the promoter region of the ROS-producing enzyme genes. Furthermore, IS inhibits antioxidant enzyme activity. The imbalance between ROS-producing enzymes and antioxidant defense renders the cell vulnerable to oxidative stress damage. AhR: aryl hydrocarbon receptor; ARNT: aryl hydrocarbon receptor nuclear translocator; IS: indoxyl sulfate; OAT: organic anion transporter; ROS: reactive oxygen species; XRE: xenobiotic response element. Parts of the figure were drawn by using pictures from Servier Medical Art. Servier Medical Art by Servier is licensed under a Creative Commons Attribution 3.0 Unported License (https://creativecommons.org/licenses/by/3.0/).

As shown in Figure 2, organic anion transporters (OATs) aid in the transcellular transport of IS across the cell membrane. OATs belong to the solute carrier 22A family, and comprise OAT1-10 and urate transporter 1. Various types of OAT are present in tissues other than kidney tissues. For example, OAT1 is also expressed in the brain, placenta, eye, liver, stomach, and olfactory epithelium [24,25]. Along with OAT3, OAT1 mediates the uptake of IS from the plasma into the cytoplasm of the target cells. In addition, OAT2 is expressed in erythrocytes, liver, and kidney tissues [26]. In these tissues, after OATs’ transportation, IS induces oxidative stress and cell damage, including in endothelial cells [27,28], vascular smooth muscle cells [29], osteoclasts [30], osteoblasts [31], erythrocytes [32], and myoblasts [33].

The aryl hydrocarbon receptor (AhR) is a ligand-activated transcription factor that participates in the biological detoxification of toxins, such as indole and its derivatives [34]. Under basal conditions, the AhR is located in the cytoplasm in an inactive form. Upon activation by IS, the AhR undergoes a conformational change that exposes its nuclear localization sequence and facilitates IS/AhR complex translocation into the cell nucleus. In the cell nucleus, this complex is able to bind to the AhR nuclear translocator (ARNT), and follows specific DNA sequences located within the promoters of target genes [35,36]. Compared with other indole derivatives, IS is the most potent ligand, due to the presence of a polar sulfate moiety that is structurally necessary for AhR activation. It is worth noting that the sulfate moiety of IS can interact with a neighboring positively charged functional group in the ligand-binding pocket of the AhR, and plays an essential role in the toxic activity associated with IS [37]. Hence, the IS/AhR/ARNT complex regulates the gene expression that underlies the key mechanism of IS-mediated toxicity observed in CKD. AST-120, an oral charcoal adsorbent, is therapeutically used to lower serum IS levels in CKD patients [22]. AST-120 adsorbs bacteria-produced indole in the gastrointestinal tract, and ameliorates IS-mediated cell damage in CKD through the suppression of oxidative stress [23].

## 3. Sources for Reactive Oxygen Species Formation

ROS are byproducts released during varieties of cell metabolism. ROS at a basal level can be used as signal molecules in regulating various physiological functions, including the cellular signaling pathway, and act as a defense system to kill environmental pathogens [38]. However, a high level of ROS generation, or a decrease in antioxidant defense, results in oxidative stress, which damages lipids, proteins, and DNA within cells. These cytotoxic effects impede cell function and lead to a variety of diseases [39].

Superoxide radicals can be generated after consumption of oxygen by several mechanisms, including the enzymatic activities of xanthine oxidase (XO) and nicotinamide adenine dinucleotide phosphate (NADPH) oxidase (NOX); they are also a liberated form of the mitochondrial electron transport chain. Hydrogen peroxide (H_2_O_2_) and hydroxyl radicals (·OH) are also key members of the ROS family, and contribute to renal injury. Two superoxide radicals can react with one another in a dismutation reaction, in which one radical is oxidized to oxygen and the other is reduced to H_2_O_2_. The dismutation reaction not only happens spontaneously, especially in acidic environments, but also arises from the catalyzation by superoxide dismutase (SOD) [40]. Unlike free radicals, H_2_O_2_ is a non-radical species that can easily lead to a free radical reaction within living organisms. In contrast to superoxides and hydroxyl radicals, H_2_O_2_ has a less reactive response, and participates in many physiological regulations. H_2_O_2_ can either be detoxified by antioxidants or further generate hydroxyl radicals through a Fenton reaction. H_2_O_2_ plays an important role in modulating redox metabolism signaling, as a second messenger and a peroxide sensor, to activate antioxidant enzymes [41]. A high H_2_O_2_ level is involved in several pathological responses, including ischemia/reperfusion (I/R) kidney injury [42], ischemic renal injury [43], systemic lupus erythematosus [44,45], hypertension [46], and diabetes [47], all of which induce renal damage.

NOX is located in the plasma membrane, and acts as a major generator of ROS in phagocytic cells by transferring one electron from intracellular NADPH to extracellular oxygen. Human NOX comprises seven isoform types, including NOX1-5, dual oxidase 1 (DUOX1), and DUOX2 [48]. NOX4 is the predominant NADPH isoform within the kidneys, and has an important pathophysiological role in renal disease [43,49,50]. Basal production of superoxides and peroxide hydrogen generation by NOX in the renal cortex and medulla can regulate water and salt reabsorption, due to their vasoconstrictive and antinatriuresis effects [51]. XO generates superoxides in the metabolism of hypoxanthine to xanthine and uric acid. Superoxides are kept at a basal level by the endogenous scavenging system under physiological conditions, but their excessive production under oxidative stress might further damage cellular function [52]. The mitochondrial electron transport chain is also an important source of superoxide production when coupled with the generation of ATP. Mitochondrial superoxides are generated non-enzymatically from the ubiquinol oxidation center of the cytochrome bc1 complex [53,54]. In general, NOX-family- and mitochondrial-mediated ROS generation has been considered to play a major role in the pathogenesis of oxidative-stress-related CKD complications [55].

In CKD patients, the activation of ROS-producing enzymes and mitochondrial dysfunction promotes oxidative burden in the vascular wall, which governs the oxidation of lipids and lipoproteins, low-density lipoprotein (LDL) carbamylation, endothelial dysfunction due to nitric oxide synthase uncoupling, and inflammatory responses that accelerate atherosclerosis [56].

## 4. The Formation of Reactive Nitrogen Species

Nitric oxide (NO) is synthesized enzymatically from L-arginine by NO synthase (NOS). The vasodilation effect of endothelial NOS (eNOS) can prevent platelet aggregation and adhesion, LDL oxidation, VSMC proliferation, and local inflammation. Oxidative stress in CKD uncouples the NOS enzyme itself to become a superoxide-generating enzyme, by transferring electrons from NOX enzymes to oxygen [57]. Thus, excessive ROS generated in CKD reduce bioactive NO production through the inhibition of NOS, and concurrently react with NO to from peroxynitrite (ONOO^-^), which is a potent RNS in terms of nitrosative stress [58,59].

RNS comprise various NO-derived compounds—including nitrogen dioxide, nitronium cations, nitrosoperoxycarbonate anions, nitryl chloride, and peroxynitrite—which can react with lipids, proteins, and DNA [60]. IS induces endothelial dysfunction by increasing both superoxide and peroxynitrite production within vascular endothelial cells, and contributes to the development of cardiovascular complications in CKD [59]. IS has therefore been recognized as an endotheliotoxin. Peroxynitrite is also involved in the pathogenesis of acute kidney injury induced by sepsis [61], I/R kidney injury [62], diabetic glomerular lesions [63], and lipopolysaccharide-induced renal dysfunction [64]. Blockade of inducible NOS (iNOS) ameliorates peroxynitrite-mediated glomerular and tubular dysfunction, and alleviates renal tubular injury biomarkers after I/R injury [62]. In addition, in the lipopolysaccharide-induced kidney injury model, inhibition of iNOS reverses the noxious effect of endotoxemia-induced decreases in renal blood flow and GFR in renal injury during sepsis [64]. Thus, excessive peroxynitrite generated by NOS plays an important role in mediating several renal diseases.

## 5. Endogenous Antioxidant Defense

There are several antioxidant systems that act as defense systems against ROS and RNS to maintain physiological function. Among them, SOD, catalase, glutathione peroxidase (GPx), glutathione reductase (GR), and glutathione *S*-transferase (GST) belong to the enzymatic system, and reduced glutathione (GSH) is a non-enzymatic system for endogenous antioxidant defense [55] (Table 1). Superoxide dismutase can convert to hydrogen peroxide in the presence of SOD, which is ultimately detoxified to oxygen and water by catalase [48]. GPx is a hydrogen peroxide scavenger, and collaborates with reduced GSH to form oxidized glutathione (GSSG), which detoxifies hydrogen peroxide into water [65]. GSSG can subsequently be reduced with the help of GR and NADPH. The ratio of GSH/GSSG is a convenient indicator by which to evaluate cellular redox status. In a resting cell, this ratio exceeds 100:1, but decreases to 10:1 or less under oxidative stress conditions [66]. Serum GSH in animal CKD is significantly lower than that of the control group, and is accompanied with increased GSSH levels. As a result, the GSH/GSSG ratio is significantly reduced in animal CKD. In addition, this ratio is in inverse correlation with the serum creatinine level, which indicates that oxidative stress is increased during CKD progression [67]. In diabetic CKD patients, a decreased GSH/GSSG ratio renders erythrocytes vulnerable to lipoperoxidation, and is associated with the apoptotic induction of erythrocytes, which leads to renal anemia [68].

Surprisingly, IS has been reported to exert antioxidant properties, balancing oxidative stress in CKD based on its physiological concentration in serum. In the presence of concentrations of less than 10 μM in human umbilical vein endothelial cells, IS showed radical scavenging ability against superoxide generation by XO in lipopolysaccharide-stimulated neutrophils [69]. Such concentrations of IS also effectively scavenge peroxyl radicals. It is especially noteworthy that IS, like SOD, serves as an endogenous antioxidant to eliminate superoxides in the blood and protect endothelial cells from oxidative burst under physiological conditions [70].

## 6. Pro-Oxidant Effects of IS in Cardiovascular Disease

**Figure 3:**
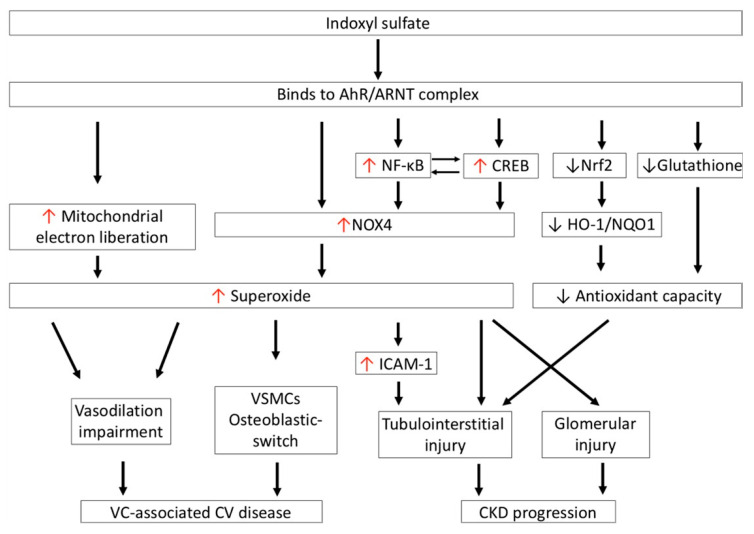
IS-induced oxidative stress is associated with cardiovascular disease and CKD progression. IS increases superoxide generation via NOX4 activation and the mitochondrial electron transport chain. Excessive superoxide generation impairs vasodilation and induces transdifferentiation of VSMCs to the osteoblastic phenotype, which is involved in the development of vascular-calcification-associated CV disease in CKD patients. In addition, IS activates NF-κB and CREB, which coordinately regulate one another to increase superoxide generation via NOX4 in proximal renal tubules; this leads to tubulointerstitial injury. IS also increases NOX-mediated superoxide generation in mesangial cells, which results in glomerular injury. Moreover, IS impairs antioxidant defense by reducing the expression of glutathione and the antioxidants HO-1 and NQO1, by downregulating transcription factor Nrf2 in renal proximal tubular cells; these aggravate tubulointerstitial injury in the progression of CKD. CKD: chronic kidney disease; CREB: cAMP response element-binding protein; HO-1: heme oxygenase-1; ICAM-1: intercellular adhesion molecule-1; IS: indoxyl sulfate; NOX4: nicotinamide adenine dinucleotide phosphate oxidase 4; NQO1: NAD(P)H quinone oxidoreductase 1; Nrf2: nuclear factor (erythroid-derived 2)-like 2; VC: vascular calcification; VSMCs: vascular smooth muscular cells.

Increased IS accumulation contributes a significant CV risk in patients with CKD and end-stage renal disease (ESRD), as summarized in Figure 3 [73]. IS significantly increases superoxide generation in endothelial cells, and attenuates vasorelaxation induced by sodium nitroprusside through the effect of the AhR and the activation of NOX [74]. The impairment of vasorelaxation after IS treatment can be reversed by a given superoxide scavenger or organic anion transporter inhibitor in tissue preparations of rat abdominal aortae [75]. Therefore, IS is considered to be a vascular toxin. Serum IS levels present a positive correlation with aortic calcification and vascular stiffness, and are a useful tool to predict overall and CV death in CKD patients [76]. Furthermore, IS also increases superoxide generation in vascular smooth muscular cells (VSMCs) in a time- and concentration-dependent manner. IS-mediated superoxide generation in VSMCs is predominantly derived from the upregulation of NOX4, but is not related to XO or to the mitochondrial electron transport chain pathway [77]. In addition, NOX4 is also involved in IS-promoted osteoblastic phenotype transition of VSMCs by increasing osteoblast-specific proteins, alkaline phosphatase, osteopontin, and core-binding factor-1 production [77]. These findings suggest that the accumulation of IS in serum during renal excretory deterioration increases the NOX4-mediated oxidative stress burden, and has a deleterious effect on endothelium-dependent vasodilatation and transdifferentiation of VSMCs to the osteoblastic phenotype, which is involved in the development of vascular calcification in CKD patients.

Furthermore, flow-mediated dilation (FMD) of endothelial cells is an indicator representative of endothelial function. Treatment with AST-120 can significantly improve FMD delay in CKD patients; that is, AST-120 has a beneficial effect on the improvement of endothelial function due to an IS-lowering effect [78]. Furthermore, AST-120 treatment for 24 months reduces carotid artery intima–media thickness, pulse wave velocity, and the risk of arterial stiffness, which are associated with carotid artery disease in CKD patients [79].

## 7. Pro-Oxidant Effects of IS in Damaged Kidneys

### 7.1. Effects on Glomerular Cells

It is well known that the clearance of IS by the kidneys is impaired in advanced-stage CKD, and leads to an elevated serum IS levels. A high serum IS level exhibits a nephrotoxic effect, which is a significant predictor of the progression rate of renal function decline and the need for of renal replacement therapy in CKD patients [80,81]. Similarly, administration of IS to diabetic mice accelerates renal damage by mesangial expansion, podocyte effacement, and glomerular basement membrane thickening, all of which contribute to albuminuria [82]. An in vitro study of IS on mesangial cells revealed that exposure of cells to IS at the concentrations seen in CKD patients would increase intracellular superoxide, hydrogen peroxide, and peroxyl radical generation through NOX activation, and lead to mesangial cell toxicity. Moreover, IS increases superoxide generation in the extracellular media from mesangial cell cultures, indicating a deleterious effect of extracellular ROS on neighboring cells in a paracrine fashion, including glomerular podocytes, endothelial cells, and inflammatory cells [83]. Notably, the antioxidant system in animal CKD is significantly diminished, as shown by the reduction in renal superoxide scavenging activity and the decrease in kidney tissue SOD activity [84].

### 7.2. Effects on Renal Tubular Cells

Organic anions, including IS, are typically eliminated into urine through the OATs expressed in proximal renal tubular epithelial cells. In humans, OATs 1–3 are mainly expressed in the basolateral membrane, while OATs 4–10 are expressed in the apical membranes of the proximal renal tubules. Basolateral uptake of IS from the peritubular capillaries is a tertiary active process that is driven by the sodium gradient and the exchange of dicarboxylic acids [85]. However, this transcellular pathway of IS delivery across the tubular cell membrane is directly cytotoxic to the renal tubules; therefore, IS is considered to be a renal tubular toxin that contributes to the development of CKD progression [81]. In a primary cell culture study, exposing human proximal renal tubular cell line HK-2 and porcine proximal renal tubular cell line LLC-PK1 to IS showed that IS directly induced cell death after cellular uptake of IS through OATs. The induction of 12-hydroxyeicosatetraenoic acid production and the subsequent increases in transient receptor potential vanilloid 1 (TRPV1) function are crucial elements in the pathogenesis of IS-mediated tubulotoxicity [86,87]. Notably, IS binding to the AhR complex in renal tubular cells also increases the expression of phosphorylation of cAMP response element-binding protein (CREB) and nuclear factor-κB (NF-κB). Activated CREB and NF-κB then increase NOX4 expression and downregulate signal transducer and activator of transcription 3 (STAT3) protein phosphorylation at tyrosine residues, which increase ROS generation in the proximal tubular cells [88]. Interestingly, ROS can positively feedback increased NOX4 expression in proximal tubular cells. ROS, NF-κB, and CREB can regulate one another, which further aggravates the oxidative stress in proximal renal tubules [81,88]. In addition, Shimizu et al. further demonstrated that IS-activated NF-κB and subsequent superoxide generation significantly increase the expression of intercellular adhesion molecule-1 (ICAM-1)—an adhesion molecule expressed in HK-2 cells and hypertensive animals that plays an important role in the pathogenesis of tubulointerstitial injury and renal fibrosis [89].

Furthermore, IS impairs the antioxidant capacity of renal tubules against oxidative stress. Glutathione levels were significantly decreased after treatment with IS at CKD concentrations in LLC-PK1 proximal tubular cells [90]. Furthermore, Bolati et al. proposed that the expression of nuclear factor (erythroid-derived 2)-like 2 (Nrf2) is downregulated in HK-2 cells, and shows less staining in rat kidney cells after IS-induced NF-κB activation. Nrf2 is a transcription factor that regulates several antioxidants and detoxifying enzymes in response to excessive amounts of free radicals [91]. Upon activation of Nrf2, it translocates to the nucleus and induces the transcriptional activation of its target antioxidant genes—such as heme oxygenase-1 (HO-1) and NAD(P)H quinone oxidoreductase 1 (NQO1)—with antioxidant response elements (AREs). HO-1 is normally expressed at a low level, and can be increased to a high level in response to oxidative stress stimuli. HO-1 is responsible for the catalyzation of the rate-limiting step of heme oxidation, and functions as an antioxidant by converting heme to carbon monoxide, ferrous iron, biliverdin-IXα, and bilirubin-IXα, which possess potent antioxidant properties against oxidative stress [92]. NQO1 is also an inducible flavoprotein enzyme, which functions as an electron reductase to produce an antioxidant form of ubiquinone and vitamin E, and at higher levels it functions as a direct superoxide reductase [93]. Thus, IS can downregulate renal Nrf2 expression and increase oxidative burden on renal proximal tubular cells.

Peroxynitrite is a powerful oxidant, and nitrifies IS to form 2-nitro-IS. Ishima et al. reported that the cytotoxicity of 2-nitro-IS to HK-2 cells is 10-fold higher than that of IS, because 2-nitro-IS induces at least several-fold intracellular ROS generations compared with IS after being transported into the cell through the help of OATs. Similarly to IS, Nrf2 activation and subsequent HO-1 induction are involved in the generation of intracellular ROS production in 2-nitro-IS-treated proximal tubular cells [94].

AST-120 not only reduces serum and urine IS levels, but also stimulates the excretion of IS from feces, which delays renal function decline in CKD through its IS-lowering effect. AST-120 alleviates oxidative stress in animal CKD kidney tissues, as evidenced by the reduction of acrolein, an end product of lipid peroxidation. The reducing ability against ROS is markedly impaired in animal CKD; after being given AST-120 for 20 weeks, the reduced SOD activity in kidney cells is greatly restored. Furthermore, AST-120 also diminishes the production of superoxides and peroxyl radicals in renal mitochondria, which are enhanced by IS. Hence, the use of AST-120 might potentially restore the antioxidant ability of kidney tissue and preserve renal function in CKD through the adsorption of indole in the gut [51].

The deleterious effect of oxidative burden is implicated in the pathogenesis of IS-induced tubular cells’ cytotoxicity, and increases the release of inflammatory cytokines and the profibrotic factor transforming growth factor β1 (TGF-β1), which mediate the complication of renal fibrosis [81]. Renal fibrosis is the adaptive response to various renal insults, and is characterized by extracellular matrix deposition, such as glomerular sclerosis, tubular necrosis, interstitial inflammation, and fibrosis, which impair glomerulus filtration and tubular function and eventually led to end-stage CKD [95,96].

## 8. Pro-Oxidant Effects of IS on Renal Osteodystrophy

Renal osteodystrophy (ROD) is a disorder that is manifested by the alternation of musculoskeletal morphology in patients with CKD. Disturbances in calcium, phosphate, parathyroid hormone (PTH), and bone metabolism are prevalent in CKD patients with an estimated glomerular filtration rate (eGFR) of less than 60 mL/min/1.73 m^2^, or even at earlier stages of CKD [97]. Renal osteodystrophy can be classified by turnover (T), mineralization (M), and bone volume (V)—known as the TMV system. Hence, ROD can be quantified by bone histomorphometry, and defined as high- or low-turnover bone disease [98]. High-turnover bone disease comprises osteitis fibrosa cystica and mixed uremic osteodystrophy. In osteitis fibrosa cystica, the activity of osteoclastic bone resorption is significantly enhanced by PTH overproduction, and is therefore coupled with osteoblast bone formation, presenting with extensive bone marrow fibrosis [99]. In contrast, low-turnover bone disease comprises adynamic bone disease and osteomalacia, which are associated with low PTH levels or PTH resistance [100]. These two diseases are both characterized by reduced osteoclast and osteoblast activity; low-to-medium bone volume and normal mineralization occur in adynamic bone disease, whereas low-to-normal bone volume and abnormal mineralization occur in osteomalacia [101,102]. Due to these different bone histomorphometric changes, low bone quantity (mass) and impaired bone quality (bone strength and toughness) increase fracture risk in CKD and ESRD patients [100,103].

**Figure 4:**
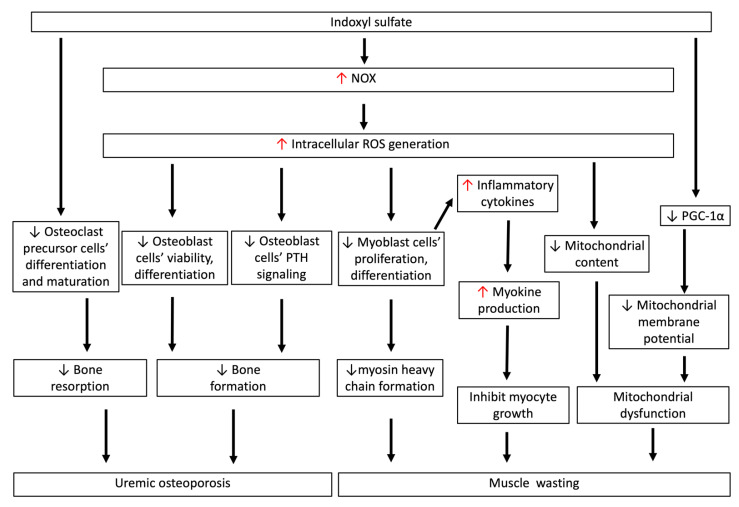
IS-induced oxidative stress is associated with uremic osteoporosis and muscle wasting. IS directly inhibits the differentiation and maturation of osteoclast precursor cells. IS inhibits cell viability and differentiation, and induces apoptotic cell death of osteoblasts through increasing intracellular ROS generation. Such ROS production also disturbs osteoblastic PTH signaling, which impedes bone formation and related mineralized matrix deposition processes. This low-turnover bone disease in early-stage CKD is characteristic of reductions in both bone resorption and formation rates, and is called uremic osteoporosis. IS-induced ROS generation directly inhibits myoblast cell proliferation and differentiation to myosin heavy chain, and also induces myokine secretion, which inhibits myocyte growth. In myoblast cells, IS-induced ROS generation reduces mitochondrial content in early CKD, and decreases PGC-1α production in myoblast cells, lowering the mitochondrial membrane potential. The decrease in myosin heavy chain, myocyte growth, and mitochondrial dysfunction contributes to the pathogenesis of muscle wasting in CKD. CKD: chronic kidney disease; IS: indoxyl sulfate; ROS: reactive oxygen species; PGC-1α: peroxisome proliferator-activated receptor gamma coactivator-1α.

As shown in Figure 4, IS has a negative impact on the differentiation of osteoclasts and osteoblasts in CKD [104]. For osteoclasts, short-term exposure (<3 days) to IS led to increased activity of osteoclast precursor cells. Nevertheless, as a result of longer term exposure to IS—for example, in CKD—the activity and maturation of osteoclast precursor cells were significantly reduced after IS bounded to the AhR in the cytoplasm [35]. In in vitro cell culture, the THP-1 macrophage can be induced by the receptor activator of nuclear factor κ-B ligand (RANKL) and macrophage colony-stimulating factor (MCSF), providing a reliable human osteoclast cell model for the study of osteoclasts [105]. Interestingly, IS treatment reduced the cell viability of THP-1 macrophages, and concurrently increased proinflammatory cytokine and ROS generation [106]. Therefore, the differentiation and maturation of osteoclasts from precursor cells are supposed to be suppressed by IS, and whether or not these are dependent on the effects of RANKL and MCSF needs further investigation.

As expected, IS also has a noxious effect on osteoblasts. IS can directly inhibit osteoblast cell viability and induce apoptosis via caspase activity. In addition, IS decreases the differentiation of osteoblasts; consequently, the production of bone-formation-associated proteins such as alkaline phosphatase, osteonectin, and type I collagen all decrease. Intracellular ROS are increased significantly in osteoblasts after treatment with IS. The reduced osteoblast cell viability could be attenuated by probenecid, an OAT inhibitor, and the antioxidant N-acetylcysteine, meaning that intracellular ROS generation in osteoblasts is involved in the pathogenesis of IS-induced cytotoxicity [31]. Notably, intracellular ROS also disturb osteoblastic PTH signaling, and impede bone formation and related mineralized matrix deposition processes. In the osteoblast cell culture, IS suppresses cyclic adenosine 3′, 5′-monophosphate (cAMP) production and causes a reduction in PTH receptor expression [107]; that is, the direct response of IS to PTH in osteoblasts causes PTH resistance, and further aggravates low-turnover bone abnormality in early CKD. The concept of uremic osteoporosis was proposed by Fukagawa et al. to explain how uremic toxins affect bone quality in early CKD [108]. As IS accumulates during renal function deterioration, together with the secretion of a Wnt signaling inhibitor from osteocytes, IS-mediated osteoblast dysfunction results in qualitative bone loss in normal bone quantities in early CKD. In advanced CKD, the dysregulation of mineral metabolism and PTH overproduction drive indolent osteoblasts into hyperfunction, which disrupts the normal bone remodeling process and results in the loss of both bone quality and quantity [101]. In sum, IS-induced oxidative stress in osteoblasts initiates the dysregulation of the bone remodeling process in early CKD, and increases the risk of bone fracture and vascular calcification thereafter.

Due to the deleterious role of IS in low-turnover bone disease in early CKD, AST-120 is a reasonable solution to improve uremic osteoporosis. Using parathyroidectomy and nephrectomy as uremic osteoporosis models, AST-120 can significantly reverse the reduction in mineral apposition and bone formation rates in CKD by histomorphometric measurements. Moreover, gene expression—obtained from proximal metaphases of the parathyroid hormone receptor—and bone formation markers such as alkaline phosphatase and osteocalcin, are all decreased in animal CKD, but can be restored after 6 weeks of AST-120 treatment [109]. Hence, AST-120 has the benefit of improving low-turnover bone disease, which is associated with IS accumulation in CKD.

## 9. Pro-Oxidant Effects of IS on Muscle Wasting in Chronic Kidney Disease

Uremia sarcopenia is defined as an imbalance between muscle protein synthesis and catabolism that reduces muscle mass and muscle strength. Uremic sarcopenia is a frequent and serious complication in CKD patients [110]. A prospective cohort study revealed that calf muscle mass and strength in walking speed performance were significantly decreased, as they had an eGFR of less than 90 mL/min/1.73 m^2^ [111]. Muscle wasting rapidly increases with CKD advancement, and is associated with poor prognosis due to its negative impact on quality of life and the associated risk of CV and overall mortality [112,113].

As shown in Figure 4, IS can directly inhibit cell proliferation and reduce myogenic differentiation to myosin heavy chain through the increase in NOX-mediated ROS generation in murine C2C12 myoblast cells. The increased number of intracellular ROS in myoblast cells can concurrently provoke the release of inflammatory cytokines (tumor necrosis factor-α, interleukin 6, and TGF-β1) in myoblast cells, which stimulate the production of myostatin and atrogin-1—myokines that function as inhibitors to suppress muscle cell growth, leading to muscle atrophy. The muscular oxidative-stress-mediated inflammation in myoblast cells can be abrogated by the AhR inhibitor CH-223191, the antioxidant ascorbic acid, or the OAT inhibitor probenecid. IS was administered chronically to the subtotal nephrectomy animal model, and the expression of inflammatory markers and myokines as described above in the gastrocnemius muscle was significantly downregulated; these findings are consistent with findings from an in vitro experiment [33]. Furthermore, Sato et al. analyzed femoral muscle tissue sections from adenine-induced CKD mice, and showed through mass spectrometry imaging that the dissolution area of the femoral muscle contained a higher intramuscular IS concentration [114].

Mitochondrial content is reduced in early CKD, and is associated with oxidative stress and chronic inflammation. Reduced peroxisome proliferator-activated receptor gamma coactivator-1α (PGC-1α) is associated with muscular atrophy in CKD because PGC-1α is a transcription coactivator for mitochondrial biosynthesis. In murine C2C12 myoblast cells, IS significantly decreases PGC-1α expression and lowers mitochondrial membrane potential. Treatment with AST-120 for 24 weeks can significantly ameliorate myostatin and atrogin-1 production in soleus and gastrocnemius muscle atrophy and alleviate IS-induced oxidative stress and related muscle atrophy. Meanwhile, AST-120 greatly improves exercise capacity, which is associated with mitochondrial dysfunction [115].

## 10. Pro-Oxidant Effects of IS on Renal Anemia

Anemia is a common complication in patients with CKD, and affects 8.4% of CKD patients with stage 3 CKD and 53.4% patients with stage 5 CKD [116]. Anemia in CKD is associated with poor quality of life, cognitive impairment, increased risk of CV disease, and overall mortality [117,118]. The mechanisms of anemia in CKD include shortening red blood cell (RBC) survival, relative erythropoietin deficiency, folate or vitamin B12 deficiency due to anorexia, functional iron deficiency due to poor dietary iron absorption, higher iron requirements during erythropoietin supplementation, and overproduction of hepcidin—a regulator that inhibits iron transport by binding to ferroportin—in a chronic inflammation state [119]. The mean lifespan duration of RBCs is decreased as CKD progresses, from 122 days in stage 1 to only 60 days in stage 5. Hence, reduced RBC numbers in early CKD require more attention, and as early as possible, in CKD patients [120].

**Figure 5:**
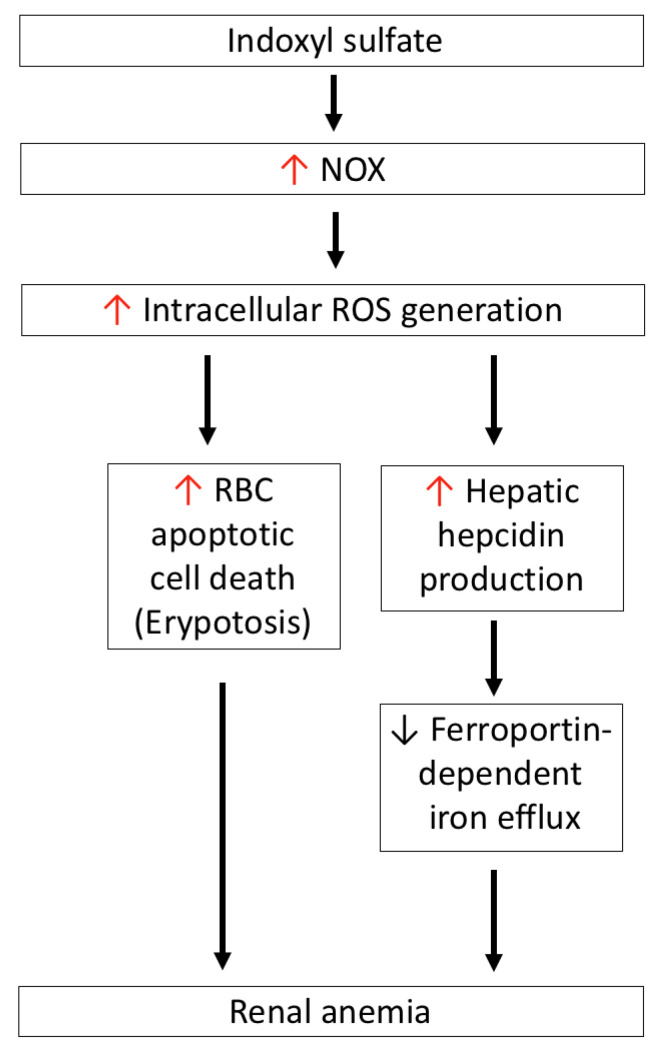
IS-induced oxidative stress is associated with renal anemia. IS-induced NOX activation mediates ROS generation, which directly induces apoptotic cell death of RBCs (eryptosis), and also increases hepatic hepcidin production to block ferroportin-dependent iron efflux from iron storage—such as duodenal enterocytes, macrophages of the reticuloendothelial system, and hepatocytes. IS: indoxyl sulfate; NOX4: nicotinamide adenine dinucleotide phosphate oxidase 4; ROS: reactive oxygen species.

As shown in Figure 5, IS induces intracellular ROS generation in RBCs in a dose-dependent manner, and concurrently induces RBC apoptotic death (eryptosis). In contrast to the expression of OAT1/3 in renal tubules, human RBC uptake of IS occurs via the help of the OAT2 transporter. After treatment with a specific OAT2 inhibitor, ketoprofen or an NOX inhibitor can abolish ROS generation and eryptosis in IS-treated RBCs. Hence, IS-induced RBC death occurs through the activation of NOX after the cellular uptake of IS by OAT2. Notably, GSH levels in IS-treated RBCs did not decrease as compared with control RBCs, which means that IS-induced RBC death occurs through a GSH-independent mechanism [32].

Hepcidin is synthesized in the liver, and tightly regulates iron homeostasis by binding to ferroportin, a cellular iron exporter that transits iron into plasma, which is abundant in duodenal enterocytes, macrophages of the reticuloendothelial system, and hepatocytes. Hepcidin blocks ferroportin-dependent iron efflux via the internalization and degradation of ferroportin in the above cells in response to intracellular or extracellular iron concentration and inflammatory mediators that decrease serum iron levels [121]. Hence, hepcidin production is proposed to be involved in the pathogenesis of anemia associated with inflammation, CKD, and some cancers [122,123]. In the HepG2 hepatic cell line, IS induces intracellular ROS generation and activates NF-κB; this is similar to what takes places in renal tubular cells and, consequently, IS participates in the induction of hepcidin expression in hepatocytes. This phenomenon can be abrogated by tempol—an ROS scavenger—the antioxidant N-acetylcysteine, or NF-κB inhibitors, but not the AhR blocker, which means that IS-induced hepcidin production occurs as a result of oxidative-stress-activated NF-κB signaling, and is independent of the AhR-mediated pathway [124]. In adenine-induced CKD mice, both serum and hepatic hepcidin levels are increased as compared to the control group, and this increase is positively correlated with serum IS levels. After CKD mice were given AST-120, the increase in hepcidin was attenuated at both the serum and hepatic levels. Furthermore, due to increased hepcidin expression in CKD mice, ferroportin in the duodenum is expectedly diminished; this can be restored by AST-120 treatment [125]. Combined with in vitro and in vivo studies, IS induces hepcidin secretion via oxidative stress, reduces ferroportin expression to delay iron efflux from iron storage, and contributes to functional iron deficiency in CKD. Concerning erythropoietin (EPO), treatment of CKD animals with AST-120 for 4 weeks can cause the decreases in mRNA and protein levels of EPO caused by IS to be restored, therefore supporting the role of IS in the development of renal anemia [126].

## 11. Clinical Studies Assessing IS and Redox Imbalance in CKD

A linear relationship exists between eGFR and renal clearance of IS, and serum IS levels are negatively correlated with eGFR (correlation coefficient = −0.7, *p* < 0.01) [127]. In a multivariate regression model, serum IS levels were proven to be associated with age, gender, eGFR, and 24-h intestinal absorption of IS [128]. Serum IS levels are progressively elevated and reach their highest levels in ESRD patients receiving maintenance dialysis. The mean value of the total serum IS level is less than 0.05–3.02 mg/L in a healthy person, and gradually increases from 1.03 at stage 1 to 4.74 mg/L at stage 4 CKD, finally increasing to 18.21 mg/L at stage 5 CKD due to impaired renal excretion [129]. Strategically, AST-120 is recognized for its effective reduction of IS levels, and subsequent alleviation of its oxidative damage to various organs in CKD patients [4,23,24].

Tomasz et al. reported that serum IS levels were positively correlated with H_2_O_2_ and Cu/Zn SOD levels, implying that IS triggers oxidative stress in CKD patients [130]. Since IS-induced oxidative stress plays an important role in the exacerbation of CKD-associated complications, lowering IS levels might provide a beneficial effect for these patients. Several studies have confirmed that the use of AST-120 decreases serum IS levels, ameliorates IS-mediated oxidative stress, and further retards the progression of CKD and its related complications. A prospective observational study showed that AST-120 treatment for 12 months in CKD patients significantly decreases urinary excretion of 8-hydroxy-2′-deoxyguanosine (8-OHdG, a DNA oxidative damage product) and L-fatty acid-binding proteins (a tubular damage marker) [23]. This observation suggests that AST-120 might alleviate IS-inducted oxidative tubular damage. In their study, AST-120 was also proven to provide effective protection against tubulointerstitial injury and proteinuria severity [23]. Shigeru et al. demonstrated that with 24 weeks of AST-120 treatment, both serum IS and reactive oxygen metabolites (dROMs) were reduced, which might protect against cardiac and renal fibrosis [131]. A crossover study conducted by Suguru et al. showed that 2 weeks of AST-120 treatment could greatly reduce serum IS levels and improve serum-oxidized albumin and 8-isoprostane (free-radical-catalyzed lipid peroxidation of arachidonate) levels in anuric maintenance hemodialysis patients [132]. A prospective observational study revealed that treatment with AST-120 for 24 weeks in CKD patients significantly decreased serum IS levels, and concurrently increased GSH to GSSG ratios, suggesting that AST-120 potentially has a direct effect on relieving oxidative stress in these patients [78]. Taken together, these studies clearly indicate that a decrease in serum IS levels as a result of AST-120 treatment reduces oxidative stress in CKD and ESRD patients.

## 12. Conclusions

The redox imbalance stands as an important mechanism underlying the IS-mediated complications in CKD, which predominantly derive from the activation of NOX and the inhibition of antioxidant capacity. In addition to serving as a pro-oxidant, IS likely exerts proinflammatory and proapoptotic effects in the pathogenesis of CKD-related complications, which merits further exploration. Although AST-120 has promising effects in terms of the prevention of the synthesis and accumulation of IS in CKD, its clinical benefits as an antioxidant are not prominent, and remain controversial. Hence, other pro-oxidant uremic toxins, including p-cresol sulfate, are noteworthy for further investigation.

## Figures and Tables

**Table 1:** Endogenous antioxidant defense.

Enzymatic Antioxidants	Mechanism
Superoxide dismutase	Metal-containing proteins that catalyze the dismutation of superoxides to hydrogen peroxide [39].
Catalase	Catalyze hydrogen peroxide to water and molecular oxygen [39].
Glutathione peroxidase (GPx)	Selenocysteine-containing residues at its active site to catalyze hydrogen peroxide to water and lipid peroxides to corresponding alcohols [58].
Glutathione reductase (GR)	Catalyze the reduction of GSSG to GSH [71].
Glutathione S-transferase (GST)	Catalyze the conjugation of the GSH to hydrophobic substrates that decrease toxicity and are predisposed to elimination from cells [72].

GSH, reduced from glutathione; GSSG, oxidized from glutathione.

## Abbreviations

8-OHdG	8-hydroxy-2′-deoxyguanosine
AhR	Aryl hydrocarbon receptor
ARNT	AhR nuclear translocator
Cbfa-1	Core binding factor-1
CKD	Chronic kidney disease
CV	Cardiovascular
eGFR	Estimated glomerular filtration rate
eNOS	Endothelial NOS
ESRD	End-stage renal disease
GPx	Glutathione peroxidase
GR	Glutathione reductase
GSH	Reduced glutathione
GSSG	Oxidized glutathione
GST	Glutathione S-transferase
ICAM-1	Intercellular expression of adhesion molecule-1
iNOS	Inducible NOS
IS	Indoxyl sulfate
LDL	Low-density lipoprotein
NADPH	Nicotinamide adenine dinucleotide phosphate
NF-κB	Nuclear factor-κB
NO	Nitric oxide
NOS	NO synthase
NOX	NADPH oxidase
OAT	Organic anion transporter
PGC-1α	Peroxisome proliferator-activated receptor gamma coactivator 1α
PTH	Parathyroid hormone
RBC	Red blood cell
ROD	Renal osteodystrophy
ROS	Reactive oxygen species
RNS	Reactive nitrogen species
SOD	Superoxide dismutase
TGF-β1	Transforming growth factor β1
VSMCs	Vascular smooth muscle cells
XO	Xanthine oxidase
ZO-1	Zonula occludens-1
